# HER2-Positive Serous Endometrial Cancer Treatment: Current Clinical Practice and Future Directions

**DOI:** 10.3390/medicina60122012

**Published:** 2024-12-06

**Authors:** Dimitrios Papageorgiou, Galateia Liouta, Ioakeim Sapantzoglou, Eleftherios Zachariou, Dimitra Pliakou, Katerina Papakonstantinou, Theofanis Floros, Evangelia Pliakou

**Affiliations:** 1 Department of Gynecology, Athens Naval and Veterans Hospital, 115 21 Athens, Greece; dimitris_papageorgiou@outlook.com (D.P.); k_papakon@yahoo.gr (K.P.); 2 Department of Medical Oncology, General Oncology Hospital of Kifissia “Agioi Anargiroi”, 145 64 Athens, Greece; lioutagalateia@gmail.com; 3 1st Department of Obstetrics and Gynecology, Alexandra Hospital, National and Kapodistrian University of Athens, 115 28 Athens, Greece; kimsap1990@hotmail.com; 4 1st Department of Gynecology, Division of Robotic and Laparoscopic Surgery, Metropolitan General Hospital, 155 62 Athens, Greece; elefthzach@gmail.com; 5 Medical School, National and Kapodistrian University of Athens, 115 27 Athens, Greece; pliakoudimitra@gmail.com; 6 5th Department of Oncology, Metropolitan General Hospital, 155 62 Athens, Greece; fanis_fl@yahoo.gr

**Keywords:** endometrial cancer, serous endometrial cancer, HER2, HER2-directed therapy, HER2-positive uterine serous carcinoma

## Abstract

The most common histological subtypes of endometrial cancer consist of endometrioid and uterine serous carcinoma, with the latter being more aggressive and accompanied by poor prognosis. Human epidermal growth factor receptor 2 (HER2) is a transmembrane tyrosine kinase receptor associated with cell proliferation, differentiation, and survival. HER2 positivity can be diagnosed in many solid tumors. It has been found that approximately one-third of the patients diagnosed with serous carcinoma may overexpress HER2/neu protein and/or show the amplification of the c-erBb2 gene. The prognostic and predictive value of HER2 biomarker is nowadays highlighted and the updates of HER2-directed treatment offer new opportunities for improved efficacy and survival. A number of HER2-targeted therapies have become available in recent years and have had promising results, prompting full drug approvals and additional investigation in many cancer types, among which is endometrial cancer. Data from clinical trials combining classical chemotherapy with anti-HER2 agents, mainly trastuzumab, alone or in combination with pertuzumab, do exist and have been incorporated into international guidelines. Moreover, further research with antibody–drug conjugates and tyrosine kinase inhibitors is being conducted. Acquired resistance remains an important problem, and its underlying mechanisms in endometrial cancer are mostly unknown. Studies exploring earlier use of Her2-directed therapy are also on the way. The purpose of this literature review is to describe the available therapies in the current clinical practice and the most prominent research data regarding the future. In any case, a number of unmet medical needs do exist for HER2-positive serous endometrial cancer, and additional research and studies are warranted to provide further understanding and improved outcomes for this tumor type.

## 1. Introduction

Endometrial cancer (EC) is the leading gynecologic cancer worldwide. In the United States alone, the American Cancer Society estimates that there will be about 67,880 new uterine cancer diagnoses and 13,250 deaths in 2024 [1]. The incidence of EC has been constantly rising, and approximately doubled over the last 20 years. The most common histological subtypes consist of endometrioid and uterine serous carcinoma (USC), with the latter being more aggressive. USC accounts for about 10% to 15% of the EC cases; nevertheless, it causes up to 40% of all EC deaths. More often, it is diagnosed at a more advanced stage, exhibits high-grade histology, and is accompanied by poor prognosis [2,3]. In earlier stages, surgery with or without adjuvant chemotherapy and radiation therapy can be curative. However, this is not feasible in advanced or recurrent tumors [2].

Human epidermal growth factor receptor 2 (HER2) is a transmembrane tyrosine kinase receptor associated with cell proliferation, differentiation, and survival. HER2 overexpression can be diagnosed in a number of solid tumors, including breast, gastric, biliary tract, bladder, pancreatic, and gynecological cancer [4]. HER2 overexpression is associated with a biologically aggressive tumor phenotype, poor prognosis, increased risk of disease recurrence, and limited benefit from chemotherapy [5,6]. HER2-directed therapy has recently become the standard of care for HER2-expressing unresectable or metastatic solid cancers, such as gastroesophageal junction and colorectal adenocarcinomas as well as non-small-cell lung cancer [7].

## 2. Biomarker Testing in Endometrial Cancer

Serous carcinoma is an aggressive histological subtype of EC as previously mentioned. The use of biomarkers in USC is emerging and it is recommended to test, among others, for the presence of microsatellite instability and HER2 overexpression [3]. It has been found that approximately one-third of the patients diagnosed with USC may overexpress HER2/neu protein and/or show the amplification of the c-erBb2 gene. In addition, some specific patient characteristics, such as Black race, are more often associated with USC and especially HER2-positive tumors [8]. Published data linking the overexpression of HER2 and/or the amplification of the c-erbB2 gene with chemoresistance and overall poor survival in USC exist, as previously mentioned; the potential prognostic role of HER2 biomarker in uterine tumors other than serous carcinoma is still under research. At the same time, the presence of HER2 positivity offers new therapeutic options for these patients and possibly better outcomes. The prognostic and predictive value of HER2 biomarkers is nowadays highlighted, and the updates of HER2-directed treatment will be discussed in this article.

Historically, HER2 amplification was the main method of assessment, first implemented over 25 years ago in HER2-positive breast cancer (BC). Nevertheless, HER2 has recently evolved into a more intricate biomarker, with intracellular kinase mutations found in breast, lung, colon, and other tumors; moreover, the epigenetic mechanisms of HER2 overexpression which are not associated with amplification have been diagnosed.

Nowadays, HER2 status in solid tumors is usually determined by HER2 immunohistochemistry (IHC). A 2+ or 3+ result on immunohistochemistry (IHC) is considered positive; for cases with any (in)complete moderate to strong membranous HER2 IHC expression, subsequent HER2 dual in situ hybridization is considered a standard of care. In a comparative analysis, a reflex testing method of restricting FISH testing only to cases that are IHC equivocal is supported [9].

Nevertheless, the HER2-low status is emerging as a new biomarker distinct from HER2-amplified disease. The first paradigm was in BC, where low levels of HER2 expression (1+ or 2+) may be adequate for delivering antibody–drug conjugates (ADCs) into the tumor environment.

On the other hand, in gynecologic malignancies, there is no standardized method of measuring HER2; gene mutations, amplifications, and overexpression are frequently diagnosed [10]. For ovarian cancer patients, next-generation sequencing (NGS) is typically performed; in EC, immunohistochemistry, mismatch repair deficiency testing, and POLE testing are now part of the staging system, and NGS is also commonly used.

Apart from that, HER2 status has not been yet researched in the setting of the molecular EC classification. However, in a study that tried to describe and correlate the clinicopathological features and prognostic significance of the HER2 status in the molecularly classified PORTEC-3 trial population, HER2 positivity was highly associated with the p53-abnormal subgroup. This study advocates that molecular characteristics rather than histology should guide biomarker testing [11].

In line with the above, the most recent National Comprehensive Cancer Network (NCCN) guidelines include HER2 IHC testing (with reflex to HER2 fluorescence in situ hybridization [FISH] for the equivocal IHC results) to be recommended for all serous and carcinosarcoma tumors. Furthermore, HER2 testing for p53-abnormal carcinomas regardless of histology is recommended to be considered [12,13,14,15,16].

## 3. HER2-Targeted Therapies

### 3.1. Trastuzumab

A number of HER2-targeted therapies have become available in recent years and have promising efficacy results, prompting full drug approvals and additional investigation in many cancer types, among which are gynecologic malignancies. There are clinical trials combining classical chemotherapy with anti-HER2 agents, mainly trastuzumab, alone or in combination with pertuzumab. Additionally, further research with ADCs and tyrosine kinase inhibitors (TKIs) is being conducted.

To begin with, trastuzumab, which inhibits tumor proliferation, is a molecule that has been used in the treatment of HER2+ BC since 1998. More specifically, it is a monoclonal antibody that binds to the extracellular subdomain IV of HER2 and has demonstrated significant benefits in progression-free (PFS) and overall survival (OS). The proposed mechanisms of action include the depletion of the surface HER2 levels on the HER2-overexpressing cells, the inhibition of the proteolytic elimination of the extracellular domain of HER2, and the augmentation of antibody-dependent cell-mediated phagocytosis (ADCP) by recruiting immune cells to the tumor. Moreover, trastuzumab suppresses the HER2-mediated cellular signaling and results in the inactivation of survival molecules such as those involved in the PI3K/Akt pathway, and the reduction in vascular endothelial growth factor (VEGF) cell levels [17,18].

In a randomized phase II study that tested the benefit of adding trastuzumab to carboplatin/paclitaxel regimen for patients with advanced, recurrent, or metastatic HER2-positive USC, the median PFS was improved in all the subgroups with an acceptable toxicity profile [12]. OS was also higher in the trastuzumab arm, while the benefit was most evident in stage III and IV disease [2].

Trastuzumab has, therefore, demonstrated clinical efficacy, safety, and a manageable toxicity profile in the treatment of HER2-positive USC, which led to its incorporation into the treatment of this aggressive tumor. According to NCCN guidelines, trastuzumab in combination with chemotherapy (carboplatin/paclitaxel) is the recommended treatment for advanced/recurrent HER2-positive USC. Recently, the carboplatin/paclitaxel/trastuzumab regimen for HER2-positive carcinosarcoma changed from category 2B to category 2A for primary or adjuvant therapy and first-line therapy in the 2/2024 updated version. This triplet regimen is recommended for patients who have not received any previous trastuzumab therapy [2,12,19].

### 3.2. Pertuzumab

Pertuzumab is another humanized monoclonal antibody that binds to HER2 on another domain; more specifically, it binds to the extracellular dimerization epitope (domain II), preventing the HER2 homo- and heterodimer formation, impeding, therefore, the activation of signaling pathways that promote cell proliferation and survival. This therapeutic option has been incorporated into the management of HER2+ disease in BC and is used in combination with trastuzumab. The rationale behind adding pertuzumab is that dual anti-HER2 therapy has the potential for further efficacy than trastuzumab alone [20].

The encouraging results of pertuzumab in other solid tumors have led to its incorporation in EC, too. In the TAPUR study, a basket trial, its combination with trastuzumab demonstrated activity in heavily pre-treated patients with EC and *HER2* amplification [21]. In another randomized phase II/III study, the primary outcome was to evaluate the use of trastuzumab and hyaluronidase-oysk or a fixed dose combination of pertuzumab, trastuzumab, and hyaluronidase-zzxf in combination with paclitaxel/carboplatin in patients with HER2-amplified USC or carcinosarcoma. The primary endpoint of the phase II study is PFS. The study is open and enrolling, and more data are awaited [22].

### 3.3. Mechanisms of Resistance

Unfortunately, although there are sufficient data to increase PFS and OS in advanced, metastatic, or recurrent HER2-overexpressing USC with the use of anti-HER2 agents, primary or secondary resistance may develop [11]. Secondary resistance remains an important problem, and its underlying mechanisms in EC are mostly understudied. Resistance to trastuzumab has been studied the most and may be attributed to a number of mechanisms, including the shedding of the HER2 receptor in the blood flow, mutations that activate the PI3K pathway, and the development of intratumoral heterogeneity of HER2 proteins [23].

In a study aimed to describe the complicated molecular alterations that happen following the anti-HER2 therapy in EC, targeted NGS, HER2 IHC, and FISH were performed on pre- and post-treatment tumor EC samples. Recurrent tumors after anti-HER2 therapy display additional genetic alterations; more specifically, the results demonstrated that the loss of target expression, more commonly, by the downregulation of expression, may serve as a mechanism of resistance to anti-HER2 therapy [23].

### 3.4. Novel Therapeutic Agents

#### 3.4.1. Lapatinib

In the landscape of HER2-positive EC treatment, orally administered TKIs have emerged as promising agents, targeting the intricate HER2 signaling pathways that are pivotal in tumor proliferation and survival. Among these, lapatinib, a dual inhibitor targeting both HER2 (ErbB2) and EGFR (ErbB1), has been extensively studied for its efficacy in many cancer types, including endometrial carcinoma. Lapatinib inhibits the intracellular tyrosine kinase domains of these receptors, preventing autophosphorylation and the subsequent activation of signaling pathways, notably the PI3K/Akt and MAPK pathways, which are important for cell proliferation and survival [24]. This inhibition is particularly effective in tumors with HER2 overexpression or amplification, which rely heavily on HER2-mediated signaling.

Research by Lin et al. [25] demonstrated that lapatinib effectively suppresses the overexpression of matrix metallopeptidase 1 (MMP1) in EC by inhibiting downstream ERK and AKT signaling pathways. Similarly, Leslie et al. [26], in a phase II trial, evaluated lapatinib’s efficacy in persistent and recurrent EC. Although the overall activity was limited in unselected cases, the identification of specific EGFR mutations, such as E690K, indicated potential benefits from lapatinib therapy in certain patients, suggesting a need for more personalized approaches in treatment.

Chu et al. [27] conducted a phase I study to determine the optimal regimen, safety, pharmacokinetics, and clinical activity of lapatinib in combination with letrozole in patients with advanced solid tumors, including EC. The combination was well tolerated and showed no pharmacokinetic interaction, demonstrating clinical efficacy with a notable partial response in an EC patient. This study underscores the importance of combination therapies in enhancing the therapeutic outcomes of lapatinib.

Further insights were provided by Engel et al. [28], who explored the interaction between MUC1, a transmembrane protein, and EGFR in EC. They found that MUC1 stimulates EGFR expression and function, enhancing cellular proliferation and survival. The knockdown of MUC1 using siRNA and CRISPR/Cas9 reduced EGFR gene expression and increased sensitivity to lapatinib. This suggests that targeting both MUC1 and EGFR could be an effective therapeutic strategy, particularly in advanced and metastatic cases where the current treatments are less effective.

The study by Groeneweg et al. [29] highlighted the potential of dual HER2 targeting with lapatinib and trastuzumab in HER2-amplified USC. This approach could significantly improve treatment outcomes by interfering with the MAPK and PI3K pathways, which are crucial for cancer cell proliferation and survival. Liu et al. [30] further supported the efficacy of lapatinib, identifying a nine-mRNA signature that included sensitivity to lapatinib, offering novel insights into the tumorigenesis and treatment of EC and providing significant clinical implications for prognosis evaluation and therapy guidance.

Despite the promising findings, resistance to HER2-targeted TKIs remains a significant challenge. Menderes et al. [31] discussed various resistance mechanisms to lapatinib in HER2-amplified USC, including the activation of alternative signaling pathways, mutations in downstream signaling molecules, and upregulation of compensatory survival pathways. They emphasized the potential of combinatorial therapies, confirming that combining HER2-targeted therapies with other treatments such as chemotherapy, other targeted inhibitors, or immune checkpoint inhibitors may enhance efficacy and overcome resistance. A review by Navarro Sanchez et al. [32] also pointed out the inconsistencies in the results of HER2-targeted therapies, including lapatinib, in USC, calling for further investigations into resistance mechanisms and combination therapies.

#### 3.4.2. Neratinib

In line with the above, the combination of neratinib and taselisib may represent a new therapeutic option for USC patients with HER2/neu gene amplification and mutated or wild-type PIK3CA, as discussed by Lopez et al. [33]. Dual HER2/PIK3CA inhibition could overcome single-agent acquired resistance, offering a promising strategy for enhancing treatment efficacy in this patient subset.

Schwab et al. [34] demonstrated the efficacy of neratinib in HER2/neu-amplified USC in vitro and in vivo as well. Their findings indicated that neratinib significantly reduced tumor growth and improved overall survival compared to control groups. Yadav et al. [35] further explored the synergistic activity of neratinib in combination with olaparib, a PARP inhibitor, showing that the combination therapy led to improved tumor suppression in HER2/neu+ USC, particularly in tumors resistant to standard chemotherapy.

#### 3.4.3. Tucatinib

Despite the advancements described above, there are currently no available data on the efficacy of tucatinib in HER2-positive EC. Future studies and clinical trials are necessary to explore the potential of tucatinib in this context and to determine its efficacy and safety profile.

In conclusion, while lapatinib and other HER2-targeted TKIs show potential in treating HER2-positive EC, challenges such as resistance mechanisms and intratumoral heterogeneity must be addressed. Combining TKIs with other therapeutic agents, refining patient selection criteria, and developing more accurate HER2 testing methods are crucial steps forward. Additionally, although current data on tucatinib in EC are lacking, its investigation in ongoing clinical trials could provide further insights into its potential role in treating HER2-positive EC. The existing literature regarding the use of TKIs in HER2+ EC are presented in Table 1.

#### 3.4.4. Antibody–Drug Conjugates

Antibody–drug conjugates constitute a promising new chapter in the therapeutic landscape for solid tumors, including HER2-positive USC. To begin with, T-DM1 has shown an antitumor effect in HER2-positive USC both in vitro and in vivo [36]. Although the existing data are compelling [37,38,39], T-DM1 may represent a different treatment option for HER2-amplified USC, refractory to trastuzumab and/or chemotherapy.

Moreover, trastuzumab deruxtecan (T-DXd), has also shown significant responses at the clinical and preclinical level in many neoplasms, including gynecological cancer. T-DXd is a novel antibody (ADC) conjugated to a topoisomerase I inhibitor that exerts its activity against HER2. It is approved and used in HER2-positive gastric and breast cancer and in HER2-mutant non-small-cell lung cancer. The benefits of T-DXd include a high drug–antibody ratio and a permeable payload through the cell membranes. This enables the transfer of its cytotoxic load to cells that do not express HER2 but are adjacent to them (bystander effect), increasing its effectiveness and possibly overcoming resistance mechanisms [40]. The clinical utility of T-DXd is confirmed by the early results of a phase II study (DESTINY-PanTumor02) which included seven pre-treated HER2 overexpressing tumor types. As for the endometrial cohort, an overall response rate of nearly 60% was demonstrated; in patients with IHC 3+ expression, the response rate was nearly 85% [7,41]. The results from the DESTINY-PanTumor02 trial have already been introduced into the NCCN guidelines, placing T-DXd in the second or subsequent lines [11].

## 4. Special Considerations for Current Clinical Practice and Future Research

The role of biomarkers as well as the potential for targeted therapies in gynecological cancers and especially, endometrial cancer are constantly expanding in oncology.

HER2 has evolved into a valuable biomarker, with implications for therapeutic decisions and novel HER2-targeted therapeutics. In addition, it is necessary to correlate molecular profiling and biomarker assessment with the treatment options [42]. The importance of HER2 testing in gynecological cancers and how to prioritize different targets in clinical practice is still debatable. The most recent validated data suggest that IHC testing for HER2 should be included in the initial diagnosis. However, the importance of standardizing testing across cancer sites and laboratories should be highlighted since it is crucial to guide and eventually optimize patient selection.

Two agents have granted, so far, approval and incorporation in the international guidelines, trastuzumab and trastuzumab deruxtecan. Trastuzumab is the NCCN compendium listed for HER2-positive USC due to the existing literature supporting clinical efficacy and an acceptable toxicity profile. Additionally, trastuzumab deruxtecan, an antibody–drug conjugate targeting HER2, also shows promise in EC and has been incorporated into the guidelines. Treatment consideration by the four molecular subtypes according to the Cancer Genome Atlas (TCGA) classification of EC is also recommended but has not been incorporated yet in the guidelines [43].

Furthurmore, since there are findings of variable HER2 expression in USC, the “HER2 low” concept is emerging following the treatment paradigm of other malignancies and could play a role in the therapeutic landscape; HER2-low tumors have a 1+ or a 2+ score on an IHC assay plus a negative result in the FISH test. In breast cancer, specific therapy options for HER2-low tumors exist, and as a result, anti-HER2 agents may be used as potential therapeutic options in EC too [44].

Apart from these, new options are urgently needed since HER2-directed agents in the recurrent, metastatic, or advanced setting are limited. Zanidatamab (ZW25), a humanized, bispecific antibody that is simultaneously attached to the two HER2 epitopes bound by trastuzumab and pertuzumab, has demonstrated safety and efficacy in HER2+ tumors in a phase 2 study; this study evaluated zanidatamab in patients with HER2-positive metastatic endometrial carcinoma/carcinosarcoma in ≥2nd line but did not meet its primary efficacy endpoint. This may be attributed to the downregulation of HER2 expression as a resistance mechanism; repeated HER2 testing should be performed prior to second-line HER2-directed therapy [45].

In that line, the DB-1303 molecule has been tested for the treatment of patients with advanced, recurrent, or metastatic EC HER2-positive therapy in the second line and beyond. DB-1303 is a third-generation ADC consisting of an anti-HER2 monoclonal antibody, a cleavable peptide-linker, and the topoisomerase I inhibitor P1003. The ADC is expected to have a wide therapeutic index, and bystander-killing effect; indeed, in HER2-positive tumor samples, DB-1303 displayed antitumor activity and safety. The agent is under evaluation in an ongoing phase 1/2a trial (NCT05150691) in patients with advanced, recurrent, or metastatic HER2-positive or HER2-low solid tumors, including EC [46].

As research continues, a number of questions arise such as the potential use of targeted therapies as a post-chemotherapy strategy in EC or as second-line options. The paradigm is moving towards the fact that the efficacy and safety of ADCs will guide treatment decisions when multiple biomarkers are present [11]. In any case, the DESTINY-PanTumor02 results call attention to the importance of HER2 expression, particularly HER2 3+ expression, in this cancer.

Studies exploring earlier use of TKIs, ADCs, or other agents in HER2-positive patients are also in the way. Moreover, research about the existing resistance mechanisms is being conducted and the results are awaited. In any case, a number of unmet medical needs do exist and additional research and studies are warranted to provide further understanding and improved outcomes.

## 5. Conclusions

Uterine serous carcinoma is usually diagnosed at a more advanced stage, exhibits high-grade histology, and is accompanied by poor prognosis. The incorporation of molecular and biomarker testing has shed some light on this aggressive subtype and offered new directions for therapeutics, especially in the HER2-positive tumors. Novel and effective agents, such as trastuzumab and T-DXd, have already been established in the clinical practice, and more data are eagerly awaited in order to introduce more options for this cancer subtype.

## Figures and Tables

**Table 1:** Overview of the existing literature regarding the use of TKIs in HER2-positive endometrial cancer.

Reference.	TKI Name	Pathway	Mechanism of Action	Results
Mano, M.S. et al. [24]	Lapatinib	HER2 signaling pathway	Inhibits HER2 and EGFR tyrosine kinase domains, preventing downstream signaling	HER2 amplification predictive of response; inconsistent with T2A aberrations
Lin, C.-Y. et al. [25]	Lapatinib	EGFR and HER2 signaling pathways	Inhibits EGFR and HER2, suppressing MMP1 expression via the ERK and AKT pathways	Effective suppression of MMP1 through EGFR and HER2 inhibition
Leslie, K.K. et al. [26]	Lapatinib	EGFR and HER2 pathways	Inhibits EGFR and HER2 tyrosine kinases, disrupting cell proliferation and survival	Limited activity in unselected cases; potential benefit with specific EGFR mutations
Chu, Q.S.C. et al. [27]	Lapatinib	HER2 and EGFR pathways	Inhibits HER2 and EGFR, disrupting cell proliferation and survival	Well tolerated with letrozole; clinical efficacy in advanced malignancies
Engel, B.J. et al. [28]	Lapatinib	MUC1 and EGFR pathways	Inhibits EGFR and HER2 tyrosine kinases, blocking tumor proliferation	MUC1 regulates EGFR; targeting both may enhance therapy
Groeneweg, J.W. et al. [29]	Lapatinib	MAPK and PI3K pathways	Inhibits HER2, blocking downstream MAPK and PI3K signaling	Promising dual targeting with trastuzumab for HER2-amplified USC
Liu, J. et al. [30]	Lapatinib	HER2 signaling pathway	Inhibits HER2 and EGFR, preventing downstream signaling	Nine-mRNA signature indicates sensitivity to lapatinib
Menderes, G. et al. [31]	Lapatinib	HER2 signaling pathway	Inhibits HER2 and EGFR, blocking the PI3K/Akt and MAPK pathways	Limited activity in USC; combinatorial therapies may overcome resistance
Navarro Sanchez, J.M. et al. [32]	Lapatinib	HER2 signaling pathway	Inhibits HER2 and EGFR, preventing downstream signaling	Challenges in efficacy; need for better HER2 testing and combination therapies
Lopez, S. et al. [33]	Neratinib and Taselisib	HER2 and PIK3CA pathways	Inhibits HER2 and the PI3K/AKT/mTOR pathways	Dual inhibition may be effective in HER2-/PIK3CA-amplified USC
Schwab, C.L. et al. [34]	Neratinib	HER2/neu signaling pathway	Inhibits HER2 and EGFR	Effective in HER2/neu-amplified USC, reducing tumor growth and improving survival
Yadav, G. et al. [35]	Neratinib	HER2/neu signaling pathway	Pan-c-erb inhibitor targeting HER2 and EGFR, enhances olaparib efficacy	Synergistic effects with olaparib in HER2/neu+ USC, improving tumor suppression

## Data Availability

Not applicable.
